# Specific heat capacity enhancement studied in silica doped potassium nitrate via molecular dynamics simulation

**DOI:** 10.1038/s41598-019-44132-3

**Published:** 2019-05-20

**Authors:** Sven Engelmann, Reinhard Hentschke

**Affiliations:** School of Mathematics and Natural Sciences, University of Wuppertal, Wuppertal, D-42097 Germany

**Keywords:** Atomistic models, Nanoparticles

## Abstract

Molten salts serve an important purpose for short term heat energy storage and as heat transfer fluids in solar power plants. Different experimental groups have shown that certain mixtures containing salts doped with small amounts of nanoparticles exhibit much greater specific heat capacities compared to the same base salts without nanoparticles. This effect is technically interesting and economically important. Thus far, however, it is not understood. Our aim is the theoretical investigation of the specific heat capacity in the aforementioned nanofluids on the molecular level using simulations. Here we present results for liquid potassium nitrate doped with silica nanoparticles. We discuss the observed increase of the specific heat in terms of the particle induced hydrodynamic reinforcement and liquid structure. The theoretical background of this discussion is a ω-space resolved phonon theory of liquids in conjunction with differential spectral densities, computed for the different systems with and without nanoparticles.

## Introduction

Nanofluids consist of solvents containing suspended nanoparticles. They latter may cause strongly altered thermophysical properties in the base liquids. These changes are sometimes quite remarkable in the sense that the effects are much greater than expected on the basis of models, which describe the properties of interests in terms of a weighted sum of the corresponding properties of the individual components^1^. Typically the nanoparticle content varies between 0.1% to 5% by weight. Among these thermophysical properties are the thermal conductivity, viscosity, or the specific heat capacity^2^. This makes nanofluids particularly interesting in heat transfer applications - especially in the context of solar thermal technologies^3,4^.

In this study we are interested in the specific heat capacity of certain types of heat transfer fluids. This thermophysical quantity, measured in units of J/gK, should be as high as possible^5^. In a previous paper^6^ one of us has given a comprehensive overview of the attendant literature. If small amounts, typically around 1% wt., of different nanoparticle, e.g., alumina^7–9^, silica and copper oxide^8^ are added to water the result is negative, which means that the studies show no increase of the specific heat capacity. In the case of molten salts as base fluids the results are quite different and significant increases of the isobaric specific heat capacity, *c*_*P*_, between a several percent to more than 20% are obtained - again for nanoparticle concentrations of around or even less than 1% wt.

Most studies focus on eutectic mixtures because of their practical significance, e.g., Li_2_CO_3_/K_2_CO_3_ doped with carbon nonotubes^10,11^, graphite nanoparticles^12^, SiO_2_^13–15^, or alumina^16^. These authors have reported specific heat capacities about 120% above the base in eutectic (62:38) mixtures of Li_2_CO_3_ with KCO_3_ containing 1.5% wt. silica nanoparticles in the range from 2 to 20 nm^17^. Another frequently studied base salt mixture is the NaNO_3_/KNO_3_ (60:40) eutectic. Again, small concentrations of alumina^18,19^, silicon dioxide^19–21^, copper and titanium^19,22,23^ as well as mixtures of silicon dioxide and alumina^19^ yield pronounced increases of *c*_*P*_. Some of these papers report that the size of the nanoparticles has a significant effect, e.g.,^17,20,21^ (Li_2_CO_3_/K_2_CO_3_ eutectic) or^18^ (NaNO_3_/KNO_3_ (60:40) eutectic). Another observation in some of the studies is that the heat capacity exhibits a maximum. Chieruzzi^19^ and Lasfargues *et al*.^22,23^ observe this maximum in the range between 0.1 to 1% wt. nanoparticle. A similar maximum is observed in ref.^24^, where the authors use the same base salt doped with Al_2_O_3_ nanoparticles. Yet another confirmation of the existence of a maximum heat capacity is found in ref.^25^. The authors study the eutectic mixture of NaNO_3_ with KNO_3_ containing silica nanoparticles in the range of concentrations from 0.5 to 2% wt. Their maximum enhancement of the heat capacity is 25%. It is important to mention that the observation of an increased heat capacity is not limited to nanofluids based on binary salt mixtures. In ref.^26^ the authors study the single component base fluid KNO_3_ containing silica and alumina nanoparticles of variable size, and observe an increase of *c*_*P*_ in the solid as well as in the liquid phase. Addition of 1% wt. nanoparticles increases *c*_*P*_ in the solid phase by between 5 to 10%. The corresponding increase in the liquid phase is roughly 6%. In ref.^27^ the authors investigate a molten ternary salt mixture. Focusing on the optimal concentration of the alumina nanoparticles, they obtain a specific heat capacity increase of about 20% at only 0.063% wt. nanoparticle concentration. An even larger enhancement of *c*_*P*_ was found in ref.^28^, where the author studies various ionic liquids containing nanoparticles. In ionic liquids the cations are large organic species and anions are organic or inorganic species. It is worth noting that the marked increases of *c*_*P*_ reported in this reference appear to be monotonic in the nanoparticle volume fraction over the entire range of volume fractions ranging up to 2.5% wt. Aside from water, molten salts or ionic liquids, other systems were studied as well. Some show no increase or even exhibit a decrease of *c*_*P*_. Examples can be found in refs^29–31^. Here the authors do find reduced specific heat capacities of nanofluids containing zinc oxide, silicon dioxide, and alumina nanoparticles, respectively, dispersed in water/ethylene glycol mixtures compared to the base fluid. Another example is ref.^32^, reporting studies on silica-ethylene glycol, silica-glycerol, and silica-glycerol/ethylene glycol (60:40) mixtures (by mass), where the nanoparticle concentration ranges from 1.0 to 4.0% wt. Still other nanofluids show a considerable increase of *c*_*P*_, for instance Zhou *et al*.^33^. They study the specific heat capacity of ethylene glycol-based CuO nanofluids and find a maximum increase of roughly 6%. Nelson *et al*.^34^ report a *c*_*P*_ of polyalphaolefin, which is increased by 50% due to the presence of graphite nanoparticles, when their mass fraction is around 0.6%.

We can summarize these results with the observation that the specific heat capacity of salt mixtures is independent of the type of nanoparticle. However, the mass concentration of particles should not be greater than 1% wt. Nanofluid preparation may vary, e.g., the temperatures during drying of the samples. But despite of this, *c*_*P*_-enhancements are between 10 to 30% on average. What is lacking thus far, however, is a molecular theory of the specific heat capacity of the above heat transfer fluids and its dependence on nanoparticle content.

Here we present the results of molecular dynamics (MD) simulations of KNO_3_ containing silica particles. We choose this system, because it exhibits an increase of *c*_*P*_ as has been shown experimentally^26,35^. A one-component base liquid is preferable, because it is simpler to parameterize as compared to a two or even multi-component base liquid. This eliminates one source of possible error. In addition, the system has already been examined using MD before^35^. We present results for the specific isochoric heat capacity, *c*_*V*_, the specific isobaric heat capacity, *c*_*P*_, as well as the isobaric expansion, *α*_*P*_, in the concentration range from 0 to 40% wt. Our suspended nanoparticle possesses a diameter of about 1.8 nm. The simulations produce a clearly discernible local maximum or ‘hump’ of *c*_*V*_ as well as of *c*_*P*_ at around 2% wt. The increase of *c*_*P*_ at the local maximum is around 3%, which is about half the increase observed in ref.^26^. In addition we observe an approximately linear increase of *c*_*V*_, which results in a 10% increase at the high end of the concentration range compared to a linear interpolation between the respective pure components, i.e., neat KNO_3_ and isolated silica particles. This suggests a second effect, aside from the one causing the ‘hump’ at low concentrations, possibly due to additional vibrational modes inside the highly structured base liquid separating the nanoparticles. Because *α*_*P*_ decreases with increasing nanoparticle concentration, we find that *c*_*P*_, aside from the ‘hump’ at low concentrations, does not exhibit such a pronounced linear increase as exhibited by *c*_*V*_ with rising particle concentration. We discuss the observed increase of the specific heats in terms of the particle induced liquid structure and the differential spectral densities, which we compute for the different systems with and without nanoparticles. The theoretical background of this discussion is the phonon theory of liquids put forward in ref.^36^.

The paper is organized as follows. The next section contains the technical details of the simulation methodology and systems studied here. Subsequently we present the simulation results including their discussion. A concise overview of theoretical concepts serving as basis for this discussion is compiled in an appendix. The final section is the conclusion.

## Results

### Specific heat capacity of the pure components

**Figure 1:**
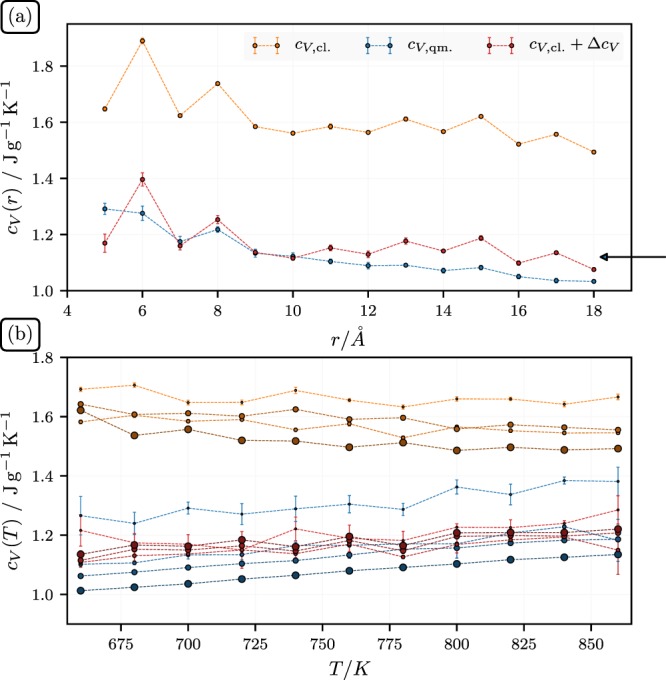
(**a**) Specific isochoric heat capacity, *c*_*V*_, of SiO_2_-particles at *T* = 700 K vs. particle radius, *r*. The arrow indicates the experimental bulk value discussed in the text, i.e., 1.12 J/gK. (**b**) *c*_*V*_ vs. *T* for four particles sizes, i.e., *r* = 5, 9, 13, and 17 Å. The size of the symbols correspond to the size of the particles.

The upper panel in Fig. 1 shows the isochoric heat capacities of isolated silica particles. The three different data sets were obtained as follows. The classical result, *c*_*V*,*cl*_, follows via

1 $${c}_{V,cl}={(m{k}_{B}{T}^{2})}^{-1}\langle \delta {E}^{2}\rangle ,$$

where *m* is the mass of the particle, *k*_*B*_ is Boltzmann’s constant, and 〈*δE*^2^〉 is the classical mean square energy fluctuation in the canonical ensemble realized using a Nosé-Hoover thermostat^37^. As a solution for the undisturbed quantum mechanical harmonic oscillator, *c*_*V*,*qm*_ is computed via

2 $${c}_{V,qm}=({k}_{B}/m){\int }_{0}^{\infty }d\omega \,g(\omega )\frac{{u}^{2}{e}^{u}}{{(1-{e}^{u})}^{2}},$$

where $u=\hslash \omega /{k}_{B}T$. The spectral distribution *g*(*ω*), obtained also from the molecular dynamics simulation, is explained in detail below. Finally, the quantum corrected classical specific heat is given by *c*_*V*,*cl*_ + Δ*c*_*V*_, where

3 $${\rm{\Delta }}{c}_{V}=({k}_{B}/m){\int }_{0}^{\infty }d\omega \,g(\omega )(\frac{{u}^{2}{e}^{u}}{{(1-{e}^{u})}^{2}}-1).$$

Notice that the first term in brackets is the contribution to the isochoric heat capacity of a harmonic oscillator with the frequency *ω* as in Eq. (2), whereas the last term in the brackets is the classical limit of this expression. Another approach utilizes ${c}_{V}=({k}_{B}/m)\sum _{i}\frac{{(\beta \hslash {\omega }_{i})}^{2}{e}^{\beta \hslash {\omega }_{i}}}{{({e}^{\beta \hslash {\omega }_{i}}-1)}^{2}}$ (*β* = (*k*_*B*_*T*)^−1^). The summation is over all normal mode frequencies calculated via a standard normal mode analysis of the particles. The resulting data are not included, as the findings are close to *c*_*V*,*qm*_ computed via Eq. (2).

Note that the reduction of *c*_*V*_ with increasing particle radius, *r* is in accord with a previous theoretical work^38^ as well as with the experimental data. A simple argument for the observed decrease of *c*_*V*_ with increasing *r* is the following. The frequency of a mode, *ω*, behaves according to *ω*^2^ = *k*/*m*_*k*_, where *k* is an average ‘spring’ constant (or stiffness) and *m*_*k*_ is a typical mass associated with this mode. The latter is limited by the linear dimension *r* of the nanoparticles. A large particle can support longer wavelength involving more mass, thus *m*_*k*_ ~ *r*. The average *k* is smaller for a small particle, because of its comparatively larger surface (where the coupling is weaker), thus *k* ~ *r*^2^ (at least approximately) and therefore *ω*^2^ ~ *r*. Thus, we expect more ‘soft’ and therefore more highly excited modes for smaller particles - mainly due to the weaker coupling at near the surface (with the exception of the silanol groups of course).

The bulk value of *c*_*V*_ for silica at *T* = 700 K is close to 1.12 J/gK, which we find via a quadratic extrapolation based on the experimental values provided in ref.^39^ at 400, 500 and 600 K. The value is in good accord with our results for *c*_*V*,*qm*_ and *c*_*V*,*cl*_ + Δ*c*_*V*_ (see also ref.^40^). The classical result is higher, as expected. Note that the Debye temperature for silicon dioxide is slightly less than 500 K in both the amorphous and crystalline states^41,42^, which is still not too far below our temperature, i.e., at 700 K we have not yet reached the Dulong-Petit limit. The bottom panel of Fig. 1 shows *c*_*V*_ vs. *T* for four particles sizes, i.e., *r* = 5, 9, 13, and 17Å. The temperature range is the range studied in this work. Notice that the upper set of classical heat capacities are close to horizontal, as they should. Their quantum counterparts, however, increase with increasing temperature, due to the increasing excitation of higher frequency modes.

**Figure 2:**
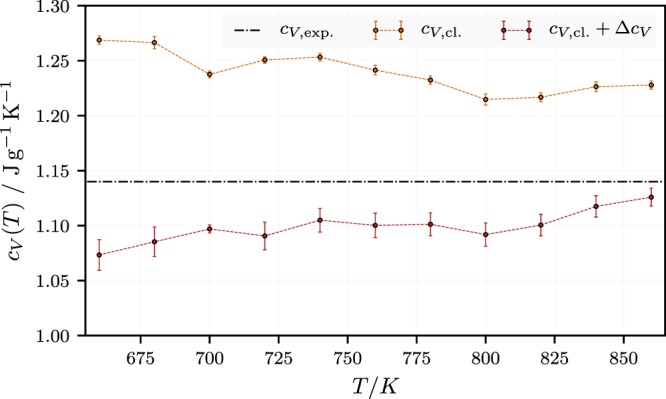
Isochoric heat capacity of neat KNO_3_ vs. temperature in the liquid phase. The horizontal line indicates *c*_*V*_ based on experimental data, which shows no clear evidence for a significant temperature dependence in this temperature range.

The following figure, Fig. 2, depicts the isochoric heat capacity of neat KNO_3_ vs. temperature in the liquid phase. Let us look to the experiments first and briefly estimate *c*_*P*_ for neat KNO_3_ via its Dulong-Petit (or high temperature) limit, i.e., *c*_*V*_ = *RN*_*f*_/(2*m*_*mol*_). *R* is the gas constant, *N*_*f*_ is the number of degrees of freedom, which here is close to 6*n*_*A*_, where *n*_*A*_ is the number of atoms per mole substance and *m*_*mol*_ is the molar mass. In the case of KNO_3_ the number of atoms is *n*_*A*_ = 5 and the molar mass *m*_*mol*_ = 101 g mol^−1^, which yields 3*Rn*_*A*_/*m*_*mol*_ = 1.23 J/gK. The corresponding experimental value is 1.14 J/gK. We calculate this number via ${c}_{V}={c}_{P}-T{\alpha }_{P}^{2}/({\rho }_{liq}{\kappa }_{T})$ from ${c}_{P}^{\,exp\,}$ above the melting point, i.e., ${c}_{P}^{\,exp\,}\approx 1.39\,{\rm{J}}/{\rm{gK}}$ in the temperature range from 620 K to 730 K^43^ (in the (older) literature ${c}_{P}^{\,exp\,}$ varies roughly between 1.35 and 1.41 J/gK (for instance^44–46^). Chieruzzi *et al*.^26^, who also study the effect of different types of nanoparticles, obtain the lowest value, i.e., *c*_*P*_ = 1.18 J/gK.). Using the values for the isobaric thermal expansion *α*_*P*_ and the liquid density *ρ*_*liq*_ from ref.^47^, i.e., 3.89 < 10^4^*α*_*P*_ < 4.07 K^−1^ and 1.778 < *ρ*_*liq*_ < 1.857 g/cm^3^ for 620 < *T* < 730 K, and the isothermal compressibility from ref.^48^, i.e., 2.1 < 10^10^*κ*_*T*_ < 2.7 Pa^−1^ for 620 < *T* < 730 K, we find $0.24 < T{\alpha }_{P}^{2}/({\rho }_{liq}{\kappa }_{T}) < 0.25$ J/gK for 620 < *T* < 730 K. We note that the density of KNO_3_ at 700 K in the simulation is found to be 1.73 ± 0.01^3^ g/cm^3^, which is less than 2% below the corresponding experimental value. In Fig. 2 we find an overall reasonable agreement with the experiment if we include the quantum corrections Δ*c*_*V*_ computed according to Eq. (3). Notice that *c*_*V*_ is almost constant, which again agrees with the experimental evidence. In the following discussion we shall frequently refer to different concepts of heat capacity in liquids, all of which are summarized in the appendix.

Firstly, it is interesting to note that generally a decrease of *c*_*V*_ with increasing temperature is observed (Here we observe a slight decrease in the uncorrected data only.). In Eq. (11) for *c*_*V*_, in the appendix, the temperature enters mainly through the two factors (1 − *r*^3^/3), where *r* is the ratio of the Frenkel frequency, *ω*_*F*_, to the Debye frequency, and (1 + *α*_*P*_*T*). The latter generally causes an increase with increasing temperature. The former, however, decreases with increasing temperature. This is because *r*∝*ω*_*F*_ and the Frenkel frequency, which really is due to a gap in momentum space rather than being a frequency gap as a recent simulation study has shown^49^, is expected to increase with increasing temperature. This in turn can give rise to the usually observed decrease of *c*_*V*_ with increasing temperature *T* in liquids. It is this explanation of the decrease of *c*_*V*_, that lends particular support to the phonon theory of liquids^50^ (cf. the extensive attendant discussion in ref.^36^). In the present case, as already mentioned, *c*_*V*_ appears to increase slightly with increasing *T* (cf. Fig. 2). But this increase is much less than what is expected based on the factor (1 + *α*_*P*_*T*).

### Specific heat capacity of the nanofluid

**Figure 3:**
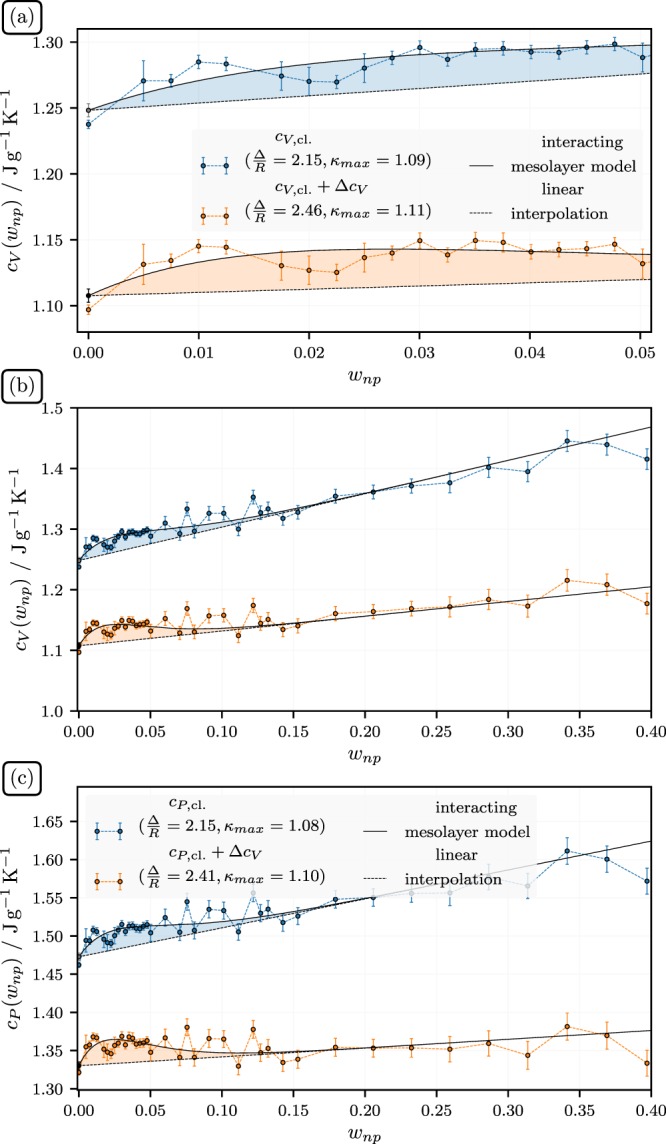
(**a**) and (**b**) Isochoric heat capacity, *c*_*V*_, of KNO_3_ doped with SiO_2_ vs. *w*_*np*_, the weight fraction nanoparticles. (**c**) Isobaric heat capacity, *c*_*P*_, vs. *w*_*np*_. The lines are discussed in the text.

The next figure, Fig. 3, presents a key result, i.e., the isochoric heat capacity, *c*_*V*_, of KNO_3_ doped with SiO_2_ vs. *w*_*np*_, the weight fraction nanoparticles, at *T* = 700 K. The upper panel shows an enlarged view of the low end of the *w*_*np*_-axis. The radius of the silica particle used here is 0.9 nm. There are two sets of data points in each panel. The upper data set is the direct result of the MD simulations, which yields the classical *c*_*V*_ via the equilibrium fluctuations of the energy in the canonical ensemble. The straight line is a linear interpolation formula connecting *c*_*V*,*liq*_ with *c*_*V*_ at around *w*_*np*_ = 0.4. Note that the data in the range $0.05\le {w}_{np} < 0.1$ fall systematically above this line. Thus, our simulations do show a small but clearly discernible increase of *c*_*V*_ beyond the linear interpolation connecting the limits of low and high *w*_*np*_. This is highlighted by the shading bounded by the linear interpolation from below and the solid line from above. The latter is a fit through the simulated *c*_*V*_-values based on the interacting mesolayer model, i.e., Eq. (14) in the appendix, developed in ref.^6^. The numerical values of the adjustable parameters, i.e., Δ/*R* und *κ*_max_, are included in the figure (here: *κ*_min_ = 1). The quantum corrections mainly cause a constant vertical scaling of the data points. Notice again that the resulting *c*_*V*_ of neat KNO_3_, including the correction, is close to its experimental value discussed above, i.e., 1.14 J/gK. The maximum of the apparent local increase of *c*_*V*_ in the doped system, beyond the linear interpolation, is close to 3%. However, the *w*_*np*_-range over which we observe this increase is larger than expected from previous experiments, which usually show no discernible effect when *w*_*np*_ is close to or greater than 0.05 (cf. ref.^6^).

It is important to note that the limiting *c*_*V*_-value for *w*_*np*_ → 1, including quantum corrections, according to the linear interpolation formula is significantly larger, i.e., 1.39 ± 0.03 J/gK, than the value expected according to Fig. 1 showing the results for isolated nanoparticles. Because the limit *w*_*np*_ → 1 is not realistic for spherical particles, it is better to make this comparison near the high end of the *w*_*np*_-range considered here, i.e., *w*_*np*_ = 0.4. But even there we find that *c*_*V*_ is 10% higher than what we expect on the basis of a linear interpolation between the respective pure components, i.e., neat KNO_3_ and isolated silica particles. This suggests a second effect, aside from the one causing the ‘hump’ in the range below *w*_*np*_ ≈ 0.1. As will be discussed below in more detail, we attribute the increase of the specific heat capacity at high *w*_*np*_ to additional vibrational modes made possible by the pronounced density modulations induced in the liquid salt between nanoparticles. However, a simple example may serve to illustrate this point. Consider a molecule adsorbing on a surface from the gas phase. This molecule gains additional heat capacity due to the ‘conversion’ of translational motion into oscillatory motion within the surface potential well.

**Figure 4:**
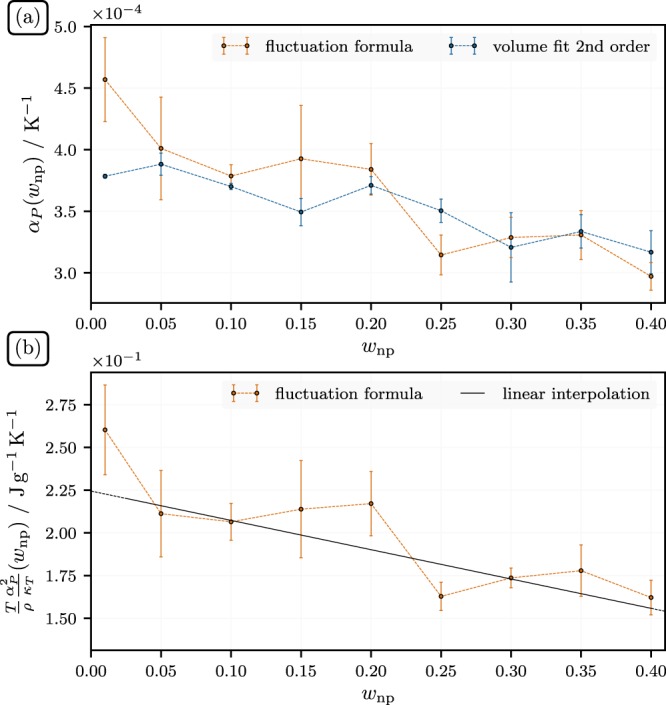
(**a**) Isobaric thermal expansion, *α*_*P*_, vs. *w*_*np*_, the weight fraction nanoparticles. (**b**) Difference between specific isobaric and isochoric heat capacity, *c*_*P*_ − *c*_*V*_, vs. *w*_*np*_. The straight line is a fit to the data used to compute *c*_*P*_ in the previous figure from the simulated *c*_*V*_-values.

**Figure 5:**
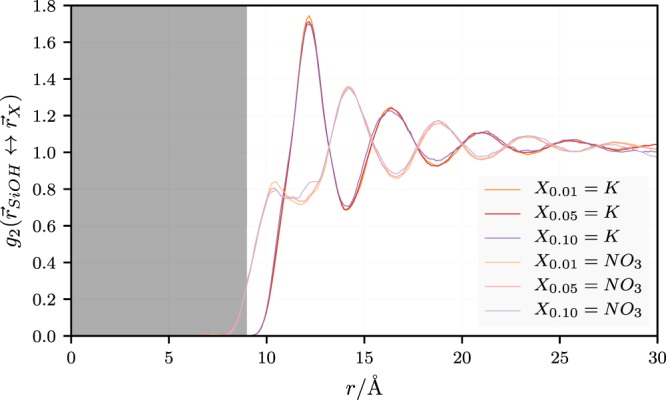
Radial distribution function *g*_2_(*r*) of silica particles with radius 9 Å obtained for the indicated weight fractions (*r* in this figure should not be confused with the particle radius). Note that the origin is the center of mass of the particle. The shading indicates the extend of the nanoparticle.

The bottom panel in Fig. 3 shows our estimate of *c*_*P*_ vs. *w*_*np*_ computed via the data for *c*_*V*_ in the middle panel in conjunction with the results for the isothermal expansion coefficient, *α*_*P*_ and $T{\alpha }_{P}^{2}/({\kappa }_{T}\rho )$ both shown in Fig. 4. Here *α*_*P*_ is computed via two distinct routes. The first one is a heating-cooling-schedule, which yields the volume at fixed *w*_*np*_ at three different temperatures including and bracketing 700 K Subsequently, *α*_*P*_ is computed on the basis of a polynomial fit through these points (the average is over 4 distinct systems). The second approach utilizes the fluctuation formula *α*_*P*_ = *k*_*B*_*β*^2^〈*δVδH*〉/*V*. The difference is largest for *w*_np_ = 0.01, but otherwise the agreement between the two methods is quite good. In order to obtain $T{\alpha }_{P}^{2}/({\kappa }_{T}\rho )$ we compute the isothermal compressibility via *κ*_*T*_ = *β*〈*δV*^2^〉/*V*. Subsequently we use a simple linear fit to these data to calculate *c*_*P*_. We note that the quantum corrected *c*_*P*_ in Fig. 3 is in good accord with the experimental result in the limit *w*_*np*_ = 0. As in the case of *c*_*V*_, we do observe the ‘hump’-like feature, exceeding the linear interpolation by 3% at its maximum. Figure 5 shows the radial pair correlation function, *g*_2_(*r*), where here *r* is the distance from the center of the particle, for the ions at different *w*_*np*_. We do observe substantial ordering imposed by the nanoparticle on the ions of the liquid. Of course, the periodicity of the system will enhance this effect, but so will the presence of neighboring particles in a true experimental situation^51^. The real difference to most experiments is the small size of the particles in our simulation, which is more than an order of magnitude less than common experimental particle sizes. In other words, in a real experiment, when the particles are well dispersed, we can expect that their separation at the same *w*_*np*_ is greater than in the simulation. Thus, on average, the surface induced ordering may be less than what is observed here (however, experimental particle sizes are average values and the widths of the attendant distributions are large, i.e., a sizable fraction of the particles may indeed be much smaller).

A useful quantity, which allows to study the influence of the nanoparticles on vibrational modes, is the spectral density *g*(*ω*), which already appeared in Eqs (2) and (3). Following ref.^52^ we compute *g*(*ω*) via

4 $$\begin{array}{rcl}g(\omega ) & = & \frac{1}{2{k}_{B}T}\sum _{j=1}^{3N}{m}_{j} {\mathcal F} \,[\langle {\dot{x}}_{j}({t}_{o}){\dot{x}}_{j}({t}_{o}+t)\rangle ](\omega )\\  & = & \frac{\pi }{{k}_{B}T}{\sum }_{j=1}^{3N}{m}_{j}\mathop{\mathrm{lim}}\limits_{\tau \to \infty }\frac{1}{2\tau }{| {\mathcal F} [{\dot{x}}_{\tau ,j}(t)](\omega )|}^{2},\end{array}$$

where

$${\dot{x}}_{\tau ,j}(t)=\{\begin{array}{ll}{\dot{x}}_{j}(t) & {\rm{for}}-\,\tau  < t < \tau \\ 0 & {\rm{otherwise}}\end{array}.$$

**Figure 6:**
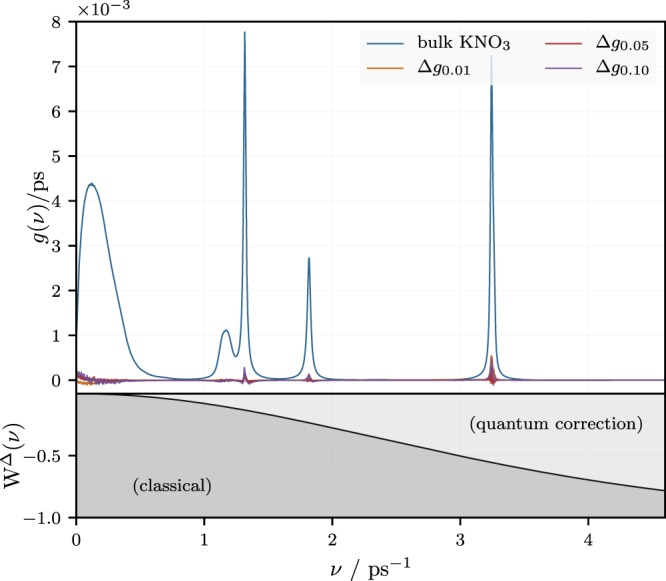
Spectral density, *g*(*ω*), vs. frequency, *ν* = *ω*/(2*π*), according to ref.^52^ obtained for neat KNO_3_ at 700 K. Also shown are difference spectra for doped KNO_3_. The same spectra are shown enlarged in the next figure. Note that *W*^Δ^(*ν*), which is the term in brackets in the argument of the integral on the right side of Eq. (3), is the statistical weight of the classical contribution to *c*_*V*_ at that frequency.

**Figure 7:**
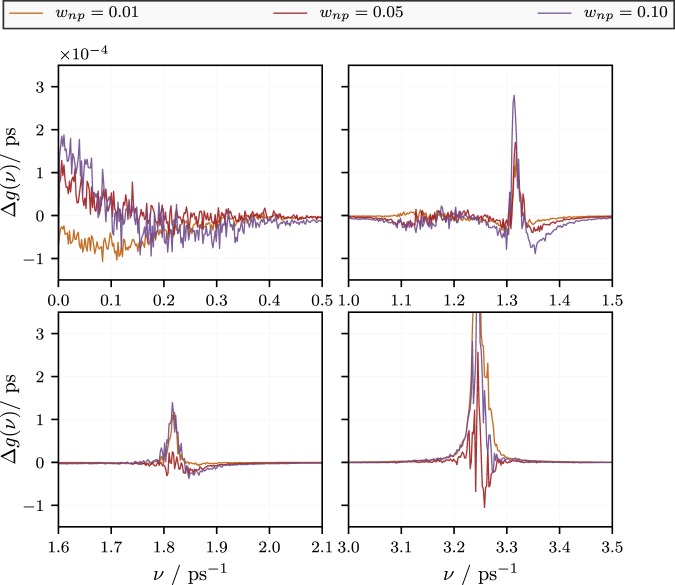
Difference spectral density, Δ*g*(*ω*), vs. frequency, *ν* = *ω*/(2*π*), following ref.^52^ obtained for doped KNO_3_. Here the spectrum for neat KNO_3_ obtained under otherwise identical conditions is subtracted from the the velocity spectra of the doped systems.

Note that ${\mathcal F} \,[\langle {\dot{x}}_{j}({t}_{o}){\dot{x}}_{j}({t}_{o}+t)\rangle ](\omega )$ is the Fourier transform of the autocorrelation function of the atomic velocity components. In particular, for *ω* = 0, this will be the diffusion coefficient. Figure 6 shows the spectral density vs. frequency, *ν* = *ω*/(2*π*), in neat KNO_3_ at 700 K. Notice the rather broad peak at small frequencies, corresponding to longer wavelength collective modes, in contrast to the rather narrow peaks at higher frequencies due to molecular modes. The effect of the presence of the nanoparticles on the spectral distribution *g*(*ω*) is best seen if we study the difference spectra ${\rm{\Delta }}g(\omega )={g}_{\mathrm{doped}}(\omega )-{g}_{{\rm{base}}\mathrm{fluid}}(\omega )$, which are shown enlarged in Fig. 7. The figure is composed of four panels, showing Δ*g*(*ω*) vs. *ν* in enlarged sections on the frequency axis of the previous figure.

We first look at the upper left panel in Fig. 7. Notice that the difference spectral density in the limit of small frequency is negative for the smallest concentration of nanoparticles, i.e., *w*_*np*_ = 0.01. For the larger concentrations, i.e., *w*_*np*_ = 0.05 and 0.1 it is positive. This indicates an excess of low frequency, i.e., classically excited, modes in the doped fluid at sufficiently high concentrations. The remaining panels show the difference spectra at the positions of the narrow peaks in Fig. 6. Here we find that the difference is overwhelmingly positive for *w*_*np*_ = 0.01. Overall this is much less the case for the higher particle concentrations.

As pointed out before, in the simulation we observe two apparent types of *c*_*V*_-enhancement. One is the ‘hump’ at low *w*_*np*_. The other one is an overall near linear increase with *w*_*np*_. Both types of of *c*_*V*_-enhancement are likely due to additional vibrational modes in excess to those present in the base liquid or the nanoparticles, individually. Additional modes can be generated by the ‘stiffening’ or hydrodynamic ‘reinforced’ of the liquid due to the nanoparticles, i.e., by a decrease of the Frenkel frequency, *ω*_*F*_. Additional modes may also occur due to the surface induced structuring of the liquid between particles, as mentioned above. Unfortunately, if we compare the *c*_*V*_-enhancement for the three particle concentrations, we notice that they differ very little. Note that we compare *c*_*V*,*cl*_ + Δ*c*_*V*_ at *w*_*np*_ = 0.01, 0.05 and 0.1 in Fig. 3 (middle panel) relative to an almost horizontal line intersecting with the vertical axis at the *c*_*V*_ of the pure salt. Figure 7 tells us that in the case of *w*_*np*_ = 0.01 the enhancement most likely is due to the frequencies around 1.8/ps and 3.25/ps. In the case of *w*_*np*_ = 0.05 and 0.1 a significant contribution is due to very low frequencies. However, notice also the following point. The experimental velocity of sound, *c*_*s*_, in KNO_3_ at about 650 K is approximately 1700 ms^−1 ^^53^. In addition, the simulation box dimension, *L*, for *w*_*np*_ = 0.05 it is about 3.8 nm, which also roughly is the distance between particles. If we take this number to be the wavelength of phonon modes, then the attendant frequency is 0.45/ps. This shows that phonon modes in the system where *w*_*np*_ = 0.05 begin to suffer frustration due to the finite system size (or, in a similar vein, due to the presence of nanoparticles). In essence this means that modes contributing to the upper left panel in Fig. 7 strongly scatter from the particles. The other panels show much more localized modes, associated with the molecular vibrations of the base fluid affected by the nanoparticles. If we carry this over to the experiments, then it is this effect of the nanoparticles on the molecular vibrations of the base fluid, which appear to be the cause for the observed enhancement of the specific heat.

## Discussion

Nanofluids, consisting of a base liquid doped with small amounts of nanoparticles (often less than 1% wt.), frequently exhibit specific heat capacities, which cannot be explained by mere additivity of the heat capacities of the constituents. In the experiments, where this has been studied, apparently the effect is the same in the solid as well as in the liquid phase. If for instance the specific heat capacity is found to increase in the liquid phase, then there is a corresponding increase in the solid phase as well. The same holds true when the specific heat capacity is diminished by adding nanoparticles. Looking at the specific heat capacity as function of nanoparticle concentration, a local maximum is observed between 0.5 to 1.5%. However, quite generally the measurements are plagued by considerable scatter - sometimes comparable to the effect itself. Surprisingly this can be true also for the pure base fluid. When nanoparticles are present, the scatter may in part be attributed to the tendency of the nanoparticles to aggregate and an attendant lack of equilibration. In the field of filled elastomers, where the underlying physics has much in common with the physics of nanofluids, this aggregation or rather flocculation is well known and the subject of extensive research^54^. One particular information, which one can carried over from rubber research, is that there is no theory thus far describing the specific interaction between the matrix material, polymers there and base liquids here, with the different types of nanoparticles. A second piece of information is the long-range hydrodynamic interaction between the particles and the surrounding matrix material.

Recently the specific heat capacity of nanofluids has been studied via molecular simulation. The authors do indeed find an enhanced heat capacity, but the reason for this is not uncovered. We also do find that SiO_2_ nanoparticles enhance the heat capacity of liquid KNO_3_ including a maximum at low nanoparticle concentrations. By studying the effect of nanoparticles of the liquid’s spectral density distribution, we conclude that the presence of the particles causes additional low frequency vibrational modes. However, these modes require high particle densities and thus cannot be responsible for the ‘hump’-like feature in the heat capacity at low particle densities. And it is this ‘hump’-like feature, which we associate with the experimentally observed specific heat capacity maximum at low particle concentrations. In addition to the aforementioned low frequency modes we find that the presence of nanoparticles enhances existing molecular modes in the base liquid. It appears that this is causing the observed ‘hump’ of the specific heat capacity. Nevertheless, it still remains unclear how exactly the particles affect the molecular modes, e.g., by distortion of the intermolecular potentials.

The differential spectral densities do not permit to probe the gap in *k*-space, which is one of the governing factors in the phonon theory of liquids. However, the heat capacity increase in doped nanofluids is present also in the solid phase. This means that the effect the nanoparticles may have on the *k*-space gap should not be the true cause of the observed heat capacity enhancement. So how about anharmonicity? Unfortunately, in this work, we do not find a significant effect of the nanoparticles on *α*_*P*_ aside from a near to linear decrease with increasing *w*_*np*_. A distortion of the intermolecular potentials should however affect *α*_*P*_. On the other hand, the effects observed in the present system are small and somewhat difficult to quantify. Future work therefore should focus on systems, which experimentally exhibit much larger enhancement of the specific heat capacity.

## Methods

### Simulation details

**Figure 8:**
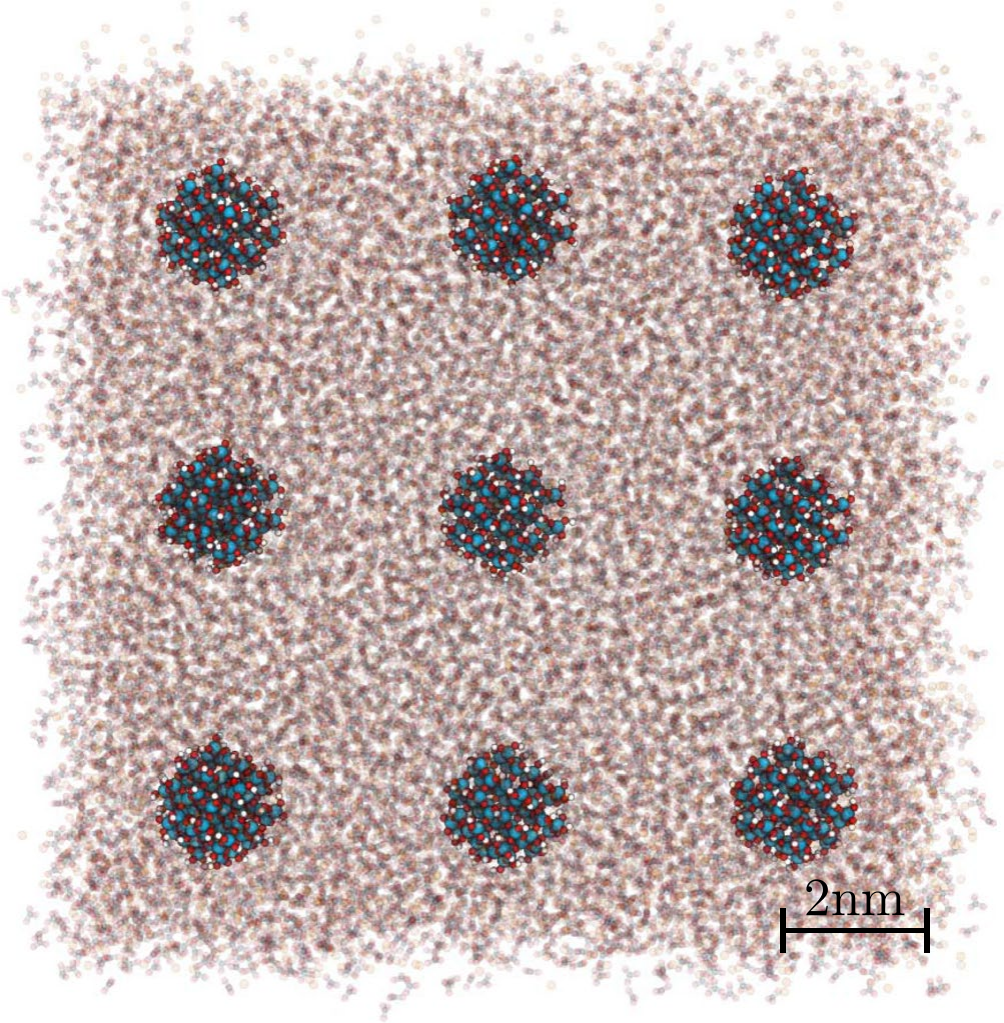
System configuration showing the actual simulation cell surrounded by its replicas corresponding to a nanoparticle weight fraction *w*_*np*_ = 0.05.

Here we use the molecular dynamics simulation technique carried out using the widely used computation package LAMMPS^55^. The force field for KNO_3_ and its parameters were taken from ref.^56^ (see also ref.^57^). All intermolecular interactions are described by a Buckingham/Coulomb potential, i.e.,

5 $${U}_{inter}({r}_{ij})={A}_{\alpha \beta }\exp (\,-\,{r}_{ij}/{\rho }_{\alpha \beta })-\frac{{C}_{\alpha \beta }}{{r}_{ij}^{6}}+\frac{{q}_{\alpha }{q}_{\beta }{e}^{2}}{4\pi {\varepsilon }_{o}{r}_{ij}},$$

with a cutoff radius of 9 Å. In the case of the Coulomb interaction long-range corrections are calculated using the PPPM method^58^ with an accuracy of 10^−6^. All bonded interactions, i.e., bond, angle and an improper dihedral potential keeping the nitrate planar, are described via harmonic potentials. Figure 8 shows an example of a typical system configuration. We model a single SiO_2_-particle immersed in liquid KNO_3_ inside a cubic volume of linear dimension *L*, applying periodic boundaries. This approach is analogous to two previous simulation studies, i.e., ref.^59^, focussing on Cu-nanoparticles in water, and ref.^35^, focussing on SiO_2_ in sodium nitrate, potassium nitrate and lithium nitrate. Nevertheless, we have considered a series of selected systems of size 2*L*, while keeping the concentration of nanoparticles constant, in order to check for finite size effects. Note that *L* and the diameter of the nanoparticle, *D*, are related via

6 $$L/D={(\frac{\pi }{6}(1+\frac{{\rho }_{np}}{{\rho }_{liq}}\frac{1-{w}_{np}}{{w}_{np}}))}^{1/3}$$

The density of the nanoparticles, *ρ*_*np*_, is roughly 2.3 g/cm^3^. The density of the base salt, *ρ*_*liq*_, is about 1.73 g/cm^3^. Finally, *w*_*np*_ is the mass fraction nanoparticles in the nanofluid. Focussing on a single nanoparticle rather than on a collection of nanoparticles immersed in a liquid, has practical reasons. Because the particle mass fractions of interest are small, modeling a collection of nanoparticles would either be prohibitively time consuming or require the particles to be unrealistically tiny. In addition, nanoparticles are known to aggregate, which probably contributes significantly to the scatter observed on the same system when it is studied by different experimental groups. This aggregation is a slow process, even on the time scale of real experiments, and inevitably causes a great deal of uncertainty in the simulations due to the limited statistics.

Our spherical SiO_2_-particles, with radii *r*, are cut from *β*-cristobalite. Subsequently the truncated valences are saturated with OH-groups. Even though industrial silica consists of amorphous particles, there is justification for this approach as has been discussed in previous work^60,61^. In the following we use the partial charges *q*_*Si*_ = 1.91, *q*_*O*_ = −0.9352, and *q*_*H*_ = 0.4238. Otherwise the silica force field is modeled according to ref.^62^, using the reparametrization procedure in ref.^63^. Interactions between the silica and the salt are modeled using the Lorentz-Berthelot mixing rules^37^. Additional details can be found in ref.^64^ as well as in the contexts of specific results.

### Selected theoretical concepts of heat capacity in liquids

**Figure 9:**
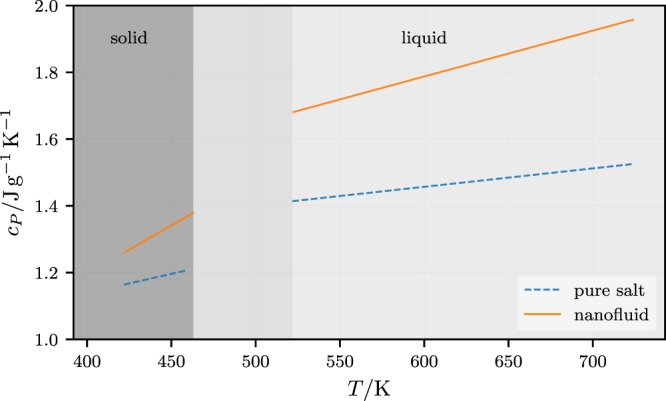
*c*_*P*_ vs. *T* for an eutectic salt mixture (NaNO_3_/KNO_3_ (60:40)) doped with SiO_2_ nanoparticles at 1% wt. from ref.^20^. Dashed lines: pure salt mixture; solid lines: salt mixture doped with nanoparticles of 60 nm diameter.

Figure 9 shows *c*_*P*_ vs. *T* for an eutectic salt mixture (NaNO_3_/KNO_3_ (60:40)) doped with SiO_2_ nanoparticles at 1% wt. (based on Figs 2 and 3 in Dudda and Shin^20^). Even though here the specific heat capacity enhancement is rather large, the figure is an overall typical example of the temperature dependence of *c*_*P*_ in the pure and in the doped system. The immediate solid-to-liquid transition region is excluded. Addition of SiO_2_ nanoparticles increases *c*_*P*_ in both phases and also increases the slope of the lines. Notice that qualitatively the solid state is not much different from the liquid state, suggesting that the underlying mechanism is at least similar in both phases.

Overall the behavior of *c*_*P*_ in the neat salt mixture is very much the same as in most common liquids, like water for instance, and even polymer melts below and above the glass transition (cf. Figure 2 in ref.^65^). The pronounced increases of the heat capacity, comparing its value on both sides of the transition (for instance 2 J/gK for water), may be understood qualitatively in terms of particles oscillating in ‘cages’. This idea dates back to Eyring *et al*. in the 1930 and was developed in the context of so-called cell models of liquids (cf. ref.^66^; chapter 4). On the solid side the molecules are more tightly constrained and corresponding oscillation frequencies are higher than on the liquid side. Therefore the Debye temperature is quite high on the solid side and (a fraction of) the vibrational modes are not fully excited below the transition temperature, resulting in a lower heat capacity of the solid phase.

Inspection reveals that the specific isochoric heat capacity of certain common liquids just above the melting transition is close to the Dulong-Petit (or high temperature) limit, i.e., *c*_*V*_ = *RN*_*f*_/(2*m*_*mol*_). *R* of course is the gas constant, *N*_*f*_ is the number of degrees of freedom, which here is close to 6*n*_*A*_, where *n*_*A*_ is the number of atoms per mole substance, and *m*_*mol*_ is the molar mass. This appears to be true for most of the base fluids in the present context.

Here we are interested in the behavior of the heat capacity far from any phase transition. At a second order phase transition, for instance, the specific heat capacity diverges due to the divergence of the fluctuation correlation length *ξ*. Note that quite generally^67^ we expect

7 $$\langle \delta e(0)\delta e(r)\rangle  \sim \exp [\,-\,r/\xi ]$$

(in the continuum limit), where *δe*(*r*) is the local energy density fluctuation at a distance *r* from the origin (in an isotropic system). At a first order transition the specific heat capacity remains finite but shows a strong temperature dependence as well. Far from a phase transition the present nanofluids typically show a weak linear *T*-dependence over the entire range of temperatures covered by the experiments. Focusing momentarily on the solid state, this observation can be explained by considering the anharmonicity of the molecular interactions. If we express the free energy *F* of the solid at high temperatures as $F={F}_{o}+3(R{n}_{A}/{m}_{mol})T\,\mathrm{ln}(\hslash \bar{\omega }/{k}_{B}T)$^68^, where the second term is the phonon contribution, then, via *E* = *F* − _*T*_∂_*T*_*F*|_*V*_, we find $E={E}_{o}+3(R{n}_{A}/{m}_{mol})T(1-T{\partial }_{T}\,\mathrm{ln}\,\bar{\omega }{|}_{V})$. Note that $\bar{\omega }$ is defined via the geometric average ${\bar{\omega }}^{3{n}_{A}}={\prod }_{i=1}^{3{n}_{A}}{\omega }_{i}$, where *ω*_*i*_ are the normal mode frequencies of the solid. It can be shown^69^ that

8 $${\partial }_{T}\,\mathrm{ln}\,\bar{\omega }{|}_{V}=-\,{\alpha }_{P}/2$$

and thus

9 $${c}_{V}\approx \frac{3R{n}_{A}}{{m}_{mol}}(1+{\alpha }_{P}T),$$

where *α*_*P*_ is considered independent of temperature. To see this we study the following two-step process in the *P*-*T*-plane. In a first step a system undergoes a thermal expansion at constant pressure, leading to a volume increase *δV*_1_ = *α*_*P*_*VδT*. In a second step the system’s volume is reduced by increasing the pressure at constant temperature, i.e., *δV*_2_ = −(*V*/*B*)*δP*|_*T*_. Here *B* is the system’s bulk modulus. We require the net volume change to vanish, i.e., 0 = *δV*_1_ + *δV*_2_ or *α*_*P*_*BδT*|_*P*_ = *δP*|_*T*_. Finally we use *V*/*B* = −*δV*/*δP*|_*T*_ once again. Defining a *δB* via *δBV* = *BδV*, we have *δB* = −*δP*|_*T*_ and thus −*α*_*P*_*BδT*|_*P*_ = *δB* or −*α*_*P*_*B* = *δB*/*δT* at constant volume. What this describes is the softening of the modulus by *δB*, which allows the volume to remain constant when the temperature increases by *δT*. The final ingredient is the assumption $B\propto {\bar{\omega }}^{2}$. On a very basic level this may be understood in terms of a harmonic oscillator for with *k* ∝ *ω*^2^, where *k* is the spring constant. Inserting this proportionality into the previous equation yields Eq. (8). To the same level of approximation as *c*_*V*_ in Eq. (9) we find for *c*_*P*_

10 $${c}_{P}\approx \frac{3R{n}_{A}}{{m}_{mol}}(1+(1+\gamma ){\alpha }_{P}T),$$

where *γ* is the Grüneisen parameter, i.e., $\gamma =V\partial P/\partial E{|}_{V}=\partial P/\partial T{|}_{V}\partial T/\partial E{|}_{V}=V{\alpha }_{P}B/{C}_{V}$. Note that (10) simply follows via *C*_*P*_ = *C*_*V*_ + *VTBα*^2^ in conjunction with (9). Finally it is worth pointing out that (9) and (10) can be obtained based on the ‘law’ of corresponding states. For instance the free enthalpy is expressed in terms of the ratio *T*/*ω*_*D*_, where *ω*_*D*_ is the Debye frequency, *G* = *G*_*o*_(*P*) + *ω*_*D*_*f*(*ω*_*D*_/*T*)^68^. From this thermodynamic potential we may calculate the heat capacities using ∂_*T*_ ln *ω*_*D*_|_*V*_ = −*α*_*P*_/2. This essentially extends the approximate validity of (9) and (10) to some extend.

Equation (9) is a limiting case of a phonon theory of liquid thermodynamics developed by Trachenko *et al*.^36,50,70^. According to its key idea, the liquid acquires solid-like behavior at frequencies exceeding a certain inverse relaxation time, the Frenkel frequency *ω*_*F*_, where the liquid may support shear waves (Recent work has shown that there is a gap in momentum space rather than a frequency gap^49^, which however does not affect the thermodynamic functions derived previously). The authors develop an energy expression of the liquid combining contributions from longitudinal phonons, transverse (shear) phonons beyond *ω*_*F*_, and diffusion below *ω*_*F*_. Expanding their result, i.e., Eq. (5) in the above reference, in the high temperature limit, neglecting again the temperature dependence of *α*_*P*_, yields

11 $${c}_{V}\approx \frac{3R{n}_{A}}{{m}_{mol}}\{(1-\frac{{r}^{3}}{3})(1+{\alpha }_{P}T)-T{r}^{2}(1+\frac{{\alpha }_{P}T}{2})\frac{dr}{dT}\},$$

where

12 $$r=\frac{{\omega }_{F}}{{\omega }_{D}}.$$

The Frenkel frequency, *ω*_*F*_, in ref.^36^ is identified with the inverse relaxation time, *τ*_*M*_, of the Maxwell model for viscoelasticity, i.e., *ω*_*F*_ ≈ 1/*τ*_*M*_ and *τ*_*M*_ = *η*/*G*_∞_. Here *η* is the viscosity and ${G}_{\infty }$ is the shear modulus at infinite frequency. In the ‘solid-limit’, i.e., *ω*_*F*_ = 0, Eq. (11) reduces to Eq. (9). In the liquid state, decreasing *ω*_*F*_ leads to an increase of *c*_*V*_. Note that every translational mode contributes *k*_*B*_/2 to the heat capacity, whereas a fully excited one-dimensional oscillator contributes *k*_*B*_. Thus, the conversion from diffusive to oscillatory motion tends to increase of *c*_*V*_. Increasing the temperature, however, leads to a decrease of *ω*_*F*_ and therefore decreases *c*_*V*_. Notice also that *dr*/*dT* > 0. The factor (1 + *α*_*P*_*T*) will counteract this to some extend, but experiments typically yield a decreasing ${c}_{V}^{\,exp\,}$^36^.

At this point we may ask how the addition of nanoparticles will affect the specific heat capacity, i.e., *c*_*V*_ as well as *c*_*P*_. As discussed previously^6^ it is not sufficient to use mass weighted averages, e.g.,

13 $${c}_{P}=(1-{w}_{np}){c}_{P,liq}+{w}_{np}{c}_{P,np}.$$

Here *w*_*np*_ is the mass fraction nanoparticles and the respective specific heat capacities of the two components are *c*_*P*,*liq*_ and *c*_*P*,*np*_. Instead it appears necessary to consider modified expressions.

One such expression is the interacting mesolayer model developed in ref.^6^. The model assumes that the center of each particle coincides with the center of a spherical shell of radius *R* + Δ, where *R* is the particle radius (in general Δ ≫ *R*). This modified version of Eq. (13) is given by

14 $${c}_{P}=[\kappa (1-{w}_{np})+(1-\kappa ){w^{\prime} }_{liq}]{c}_{p,liq}+{w}_{np}{c}_{P,np}.$$

with

15 $$\kappa ={\kappa }_{max}Y+{\kappa }_{min}(1-Y),$$

16 $${w^{\prime} }_{liq}=\frac{{\rho }_{liq}}{\bar{\rho }}Y$$

and

17 $$Y=\exp [-\frac{\bar{\rho }}{{\rho }_{np}}{w}_{np}{(1+\frac{{\rm{\Delta }}}{R})}^{3}],$$

$\bar{\rho }={\rho }_{np}{w}_{np}+{\rho }_{liq}(1-{w}_{np})$ (Note that Eqs (15) and (17) are the corrected versions of the mistyped Eqs (15) and (17) in ref.^6^). Here *κ* is the specific heat capacity in the mesolayer divided by the specific heat of the neat liquid. Note that *c*_*P*_(*w*_*np*_ = 0) = *c*_*p*,*liq*_ and *c*_*P*_(*w*_*np*_ = 1) = *c*_*P*,*np*_, i.e., Eq. (14) agrees with (13) in these limits. In between, however, Eq. (14) deviates from the linear interpolation. Increasing *w*_*np*_ from zero in the interacting mesolayer model yields a *c*_*P*_ above the linear interpolation, when *κ*_*max*_ > 1. According to the underlying idea, the heat capacity in a liquid shell of thickness Δ is enhanced due to the presence of the central particle. A further increase of *w*_*np*_ leads to a statistical overlap of the mesolayers surrounding each particle, which means that *Y* decreases from one to zero and *κ* changes from *κ* = *κ*_*max*_ to *κ* *=* *κ*_*min*_. Notice that *max* and *min* refer to the che case when the mesolayers are strongly overlapping, which may affect the heat capacity inside the mesolayer. The model, however, does not specify the microscopic origin of these changes. Nevertheless, in ref.^6^ it is shown, that it fits the experimental data quite well. Here we employ Eq. (14) to *c*_*P*_ as well as to *c*_*V*_. At this point we return to possible microscopic reasons for the change of the specific heat capacity in the presence of nanoparticles.

It is well known^71–73^ that adding particles to a liquid causes its viscosity to increase. This hydrodynamic ‘reinforcement’, which indeed is a long-range effect, should thus decrease *ω*_*F*_. This is because *ω*_*F*_ ∝ 1/*η*, resulting in an increase of *c*_*V*_ (as well as *c*_*P*_). Nevertheless, it is known that the addition of particles to an elastic matrix, usually an elastomer matrix, will increase its shear modulus^74^. Overall there is no net effect on *τ*_*M*_ = *η*/*G*_∞_. However, the Maxwell model is an oversimplification and therefore this may not hold in real system governed by relaxation time distributions rather than a single one.

It is interesting to mention that polymer melts and the liquids discussed here are quite similar. In particular, the addition of nanoparticles does affect *c*_*P*_ in a polymer melt beyond what one expects on the basis of Eq. (13). This remains true even if the polymer is vulcanized, i.e physically cross linked. In ref.^65^ the authors observe a pronounced decrease of *c*_*P*_ when small amounts carbon nanotubes are added, i.e., 1% wt. and 5% wt., both below and above the glass transition.

In summary, the effect of nanoparticles on the heat capacity, as well as on other physicochemical properties, of liquids is not colligative, i.e., it does not merely depend on the nanoparticle weight fraction. Even qualitatively it depends on particle type and size, possibly morphology. In other respects it is basic and almost universal, i.e., it affects the liquid and the solid phase alike and it is observed over a wide range of base fluids of small molecules as well as polymers. The above theoretical framework allows us to focus on three aspects - the generation of additional shear modes entering through the Frenkel frequency, anharmonicity of the intermolecular potentials, and particle (network) induced liquid structure.

## Data Availability

The datasets generated during and analysed during the current study are available from the corresponding author on reasonable request.
